# Malaria elimination in Zanzibar: where next?

**DOI:** 10.11604/pamj.supp.2023.45.1.39804

**Published:** 2023-06-18

**Authors:** Mohamed Haji Ali, Jovin Kitau, Abdullah Suleiman Ali, Abdul-wahid Al-Mafazy, Sisay Gashu Tegegne, Omar Ussi, Christine Musanhu, Shija Joseph Shija, Bakari Omar Khatib, Humphrey Mkali, Sigsbert Mkude, Geofrey Makenga, Elizabeth Kasagama, Fabrizio Molteni, Noela Kisoka, Chonge Kitojo, Naomi Serbantez, Erik Reaves, Zabulon Yoti

**Affiliations:** 1 Zanzibar Malaria Elimination Programme, Ministry of Health, Zanzibar, Tanzania,; 2 World Health Organization, Country office, Dar-es-Salaam, Tanzania,; 3 Ministry of Health, Zanzibar, Tanzania,; 4 Second Vice President Office-Zanzibar Country Coordinating Mechanism, Zanzibar, Tanzania,; 5 Population Services International, Dar-es-Salaam, Tanzania,; 6 Swiss Tropical and Public Health Institute, Allschwil, Switzerland,; 7 US President´s Malaria Initiative, United States Agency for International Development, Dar-es-Salaam, United Republic of Tanzania,; 8 United States Centers for Disease Control, Dar-es-Salaam, Tanzania

**Keywords:** Malaria, elimination, malaria programme review, Zanzibar

## Abstract

In 2018, Zanzibar developed a national malaria strategic plan IV (2018-2023) to guide elimination of malaria by 2023. We assessed progress in the implementation of malaria activities as part of the end-term review of the strategic plan. The review was done between August and October 2022 following the WHO guideline to assess progress made towards malaria elimination, effectiveness of the health systems in delivering malaria case management; and malaria financing. A desk review examined available malaria data, annual work plans and implementation reports for evidence of implemented malaria activities. This was complemented by field visits to selected health facilities and communities by external experts, and interviews with health management teams and inhabitants to authenticate desk review findings. A steady increase in the annual parasite incidence (API) was observed in Zanzibar, from 2.7 (2017) to 3.6 (2021) cases per 1,000 population with marked heterogeneity between areas. However, about 68% of the detected malaria cases were imported into Zanzibar. Malaria case follow-up and investigation increased from <70% in 2017 to 94% and 96% respectively, in 2021. The review noted a 3.7-fold increase of the health allocation in the country’s budget, from 31.7 million USD (2017/18) to 117.3 million USD (2022/23) but malaria allocation remained low (<1%). The varying transmission levels in the islands suggest a need for strategic re-orientation of the elimination attempts from a national-wide to a sub-national agenda. We recommend increasing malaria allocation from the health budget to ensure sustainability of malaria elimination interventions.

## Introduction

There have been considerable gains in the fight against malaria globally. Despite the gains, a total of 627,000 deaths and 241 million malaria cases were recorded in 2020 alone [1]. The WHO African region accounted for 95% of the global malaria cases and 96% of the deaths. In 2009, the Zanzibar Ministry of Health (MoH) conducted a systematic assessment of the possibility of eliminating malaria in Zanzibar [2]. Malaria elimination was deemed feasible in Zanzibar, albeit challenging [3]. The assessment provided recommendations to achieve and maintain a malaria-free status including: i) an optimal coverage of indoor residual spraying (IRS) and or long-lasting insecticidal nets (LLINs), ii) the establishment of a surveillance system to rapidly detect a high proportion of new malaria infections, iii) a passive case detection system through health facilities to detect and treat imported infections to minimize further transmission, and iv) substantially minimizing the risk of malaria importation in the islands. The assessment called for; v) intensification of education and communication campaigns to enhance the provision of early diagnosis and effective treatment services, and vi) training of the malaria program health workforce to manage and implement the interventions required for elimination [3]. With the fulfillment of most of the assessment recommendations, the MoH supported by WHO conducted a comprehensive malaria audit in 2015 to record progress made and further guide malaria activities in Zanzibar [4]. Following this audit and recommendations, malaria was categorized as a notifiable disease; the surveillance system was upgraded to cover all health service delivery points. Detected and treated malaria cases were characterized, classified and followed up to their areas of residency to identify any more clusters of infections and the existence of breeding habitats. Any identified clusters of cases with breeding areas were characterized as transmission foci to which appropriate responses were mounted with intense vector control interventions.

The achievements made raised optimism hence the development of the Zanzibar Malaria Strategic Plan (MSP)-IV, 2018-2023 to advance the elimination agenda by 2023 [5]. The plan aligned its strategies to the Global Technical Strategy for Malaria (GTS) 2016-2030 [6]. The strategic plan had objectives to ensure quality assured diagnosis and appropriate case management in all health facilities and communities, increase appropriate vector control measures for the population at risk of malaria, and reinforce surveillance for malaria elimination [6]. We assessed progress made between 2018 and 2022 in an attempt to eliminate malaria in the Islands, specifically in terms of the epidemiological and entomological impact and the effectiveness in the delivery of case management, surveillance, and vector control services.

## Methods

**Setting:** Zanzibar is an archipelago on the East African coast, some 50 kilometres off mainland Tanzania. Zanzibar has two main islands, Unguja and Pemba, and several smaller ones. Zanzibar has a population of 1,889,773 people; about 69% live in Unguja and 31% in Pemba Islands [7]. Zanzibar has five administrative regions and 11 districts, seven in Unguja and four in Pemba. Shehia is the lowest administrative unit in Zanzibar, with a population between 2,000 and 5,000 people. Currently, Zanzibar has a total of 388 Shehias. The climate is tropical, with two rainy seasons: long rains (March to May), and short rains (mid-October to December) with April and May being the wettest months. Total annual rainfall is about 1,600 millimetres in Unguja and 1,900 mm in Pemba.

**Malaria activities Overall management:** the Zanzibar Malaria Elimination Programme (ZAMEP) is the MoH Zanzibar organ responsible for the implementation of malaria elimination activities. The ZAMEP is made up of six technical units: i) vector control, ii) case management, iii) surveillance, monitoring and evaluation, iv) commodities and logistics management, v) Social Behaviour Change and Communication (SBCC), and vi) program management, finance and logistics.

**Case management (diagnosis and treatment) services:** three levels of public health facilities exist in Zanzibar: (a) primary level (Primary Health Care Units (PHCUs) and (b) Primary Health Care Centers (PHCCs), and (c) Secondary level (District and Regional Hospitals) and one tertiary level (Mnazi Mmoja referral and specialised hospital). All operational health facilities provide malaria diagnostic and curative services at no cost to the beneficiaries. Suspected malaria cases are tested for parasites using rapid diagnostic tests and microscopy services in health facilities with formal laboratory services. If positive, treatment is with Artesunate-amodiaquine (ASAQ), as the first-line antimalarial for uncomplicated malaria and artesunate injection if severe. At the primary level, the PHCUs and PHCCs provide pre-referral management of severe diseases with parenteral antimalarials. The referred severe malaria cases are admitted at the district and regional hospitals which are equipped to provide intravenous treatments and further management of complications.

**Vector control services:** vector control is done in collaboration with implementing partners. Currently, insecticide residual spraying (IRS) and long-lasting insecticidal nets (LLINs) are the principal vector control measures in Zanzibar, supported by the US President´s Malaria Initiative (PMI) and the Global Fund to Fight Aids Tuberculosis and Malaria (GFATM). Long-lasting insecticidal nets are distributed in two modalities, the continuous distribution targeting pregnant women and children (i.e., infants) in antenatal care (ANC) and mass replacement campaigns done every 3 years. The IRS is implemented through targeted application in malaria transmission foci with organophosphate (pirimiphos-methyl, actellic) and neonicotinoid (clothianidin). The IRS was performed in targeted areas (Shehia) with annual malaria incidence of >3.4 cases/1,000 population. From 2020, IRS is being conducted as a response intervention following notification of three local cases per week and entomological investigation.

**Procurement and distribution of commodities:** Central Medical Stores is responsible for the procurement, receipt, storage, and distribution of malaria commodities, including antimalarials, rapid diagnostic kits, microscopes and supplies and mosquito bed nets. The Central Medical Stores has a supply system to ensure commodities requested from health facilities are timely delivered to the service points.

**Health information system/District Health Information System (DHIS) and data capture:** the ZAMEP obtains malaria data through routine systems. All health facilities collect data on malaria as part of the general health services at outpatient departments (OPDs), in-patient admissions and antenatal care using weekly disease surveillance forms and entered into a web-based DHIS2 system. Malaria suspects, diagnosed and treated patients in health facilities are recorded on a daily basis. Data is summarised and used to compile and report through the electronic integrated disease surveillance and response (eIDSR). This data is submitted as part of the DHIS2 monthly reports. Additionally, passive malaria cases are collected and reported through Malaria Case Notification (MCN). The MCN data is collected at health facilities and entered using android mobile phones and tablets onto a central database. An index case is notified to a Council Malaria Surveillance Officer (CMSO), who receives (accepts), and then collects surveillance data as part of a reactive case follow-up and detection at households of index cases. The coconut surveillance system links to MCN and the reactive case follow-up data. Coconut Surveillance is an open-source mobile application system modified from the malaria early epidemic detection system (MEEDS) to support individual case reporting [8]. Through tablet Geographical Positioning System (GPS) capability, the Coconut system provides real-time case identification, and geo-located data for fine targeting of interventions. Each household member is tested, and new cases are treated immediately. Data are synchronised at least daily with a shared cloud database and data validation is done every 6 months.

**Assessment of progress in malaria activities:** the malaria strategic plan (NSP) 2018-2023 aimed to eliminate malaria in Zanzibar by 2023. Therefore, an assessment of progress in implementing malaria activities was done as part of the malaria programme review (MPR) that aimed to appraise the country´s malaria situation and programme performance for better results and impact. The MPR was conducted following the WHO guidance [9]. Before the review, comprehensive data tracing from several sources was performed in collaboration with the Swiss Tropical and Public Health Institute. A malaria profile was then developed including the outputs of the analysed data [10]. The MPR was done in three stages, desk review, external validation, and reporting. During the review, thematic teams were constituted corresponding to the six ZAMEP thematic units as indicated under the “overall management” section above. The review teams comprised the Heads of Units and staff from the respective Units, MoH staff and implementing partners supporting malaria activities in Zanzibar. To enrich the discussions, each thematic team had at least one member from the implementing partner projects from mainland Tanzania. The teams reviewed malaria data reported between 2018 and 2022 in the different systems, including DHIS2, MCN and eIDSR. Other documents reviewed by the teams for evidence of implemented malaria activities included annual work plans and annual implementation reports against the performance framework following the set guidance [9].

The review assessed progress made by the ZAMEP towards the epidemiological (malaria prevalence and parasite incidence) and entomological (vector species and sporozoite rates) impact targets of the NSP 2018/19-2022/23. Furthermore, it assessed the programme´s capacity to implement planned activities; the effectiveness of the health systems in delivering malaria case management, surveillance and vector control services; and malaria financing. This paper reports on progress made on the epidemiological and entomological impact and the effectiveness of delivering case management, surveillance, and vector control services. A team of seven external experts from malaria programmes, different institutions and WHO supported the validation stage, one corresponding to each of the ZAMEP thematic Units and one additional for multisectoral partnerships. The experts worked closely with the ZAMEP staff to validate findings from the desk review guided by pre-structured checklist and questionnaires. The validation involved visiting selected health facilities and communities and conducting interviews with key regional and council health management teams, community leaders and inhabitants to authenticate the malaria activities and services provided. The experts also held discussions with the pre-identified officials in government ministries and agencies to get their views on the implementation of malaria activities in order to validate the desk review findings. Subsequently, external experts and thematic team leads convened to consolidate findings from the desk review and external validation into the final MPR report.

**Data management and analysis:** data from MCN, IDSR/MEEDS, DHIS2 and other dedicated malaria information systems were exported into MS Excel for handling and storage. Data cleaning and analysis were done in MS Excel. Descriptive analysis was done to summarise variables along different programmatic indicators. The Plasmodium falciparum parasite prevalence, annual parasite incidence and individual malaria cases were aggregated to obtain district, regional and national estimates.

**Ethical considerations:** the malaria programme review is a routine appraisal activity done halfway through (i.e., mid-term review) or towards the end of the strategic plan implementation period (i.e., end-term review) as part of the programme strengthening process. Approval for the review was obtained from the Zanzibar MoH and regional and district-level government officials and stakeholders. Regional and council health management officials and stakeholders assented to the review. Government officials and stakeholders consented before the validation visits and discussions with the external reviewers.

## Results

**Figure 1:**
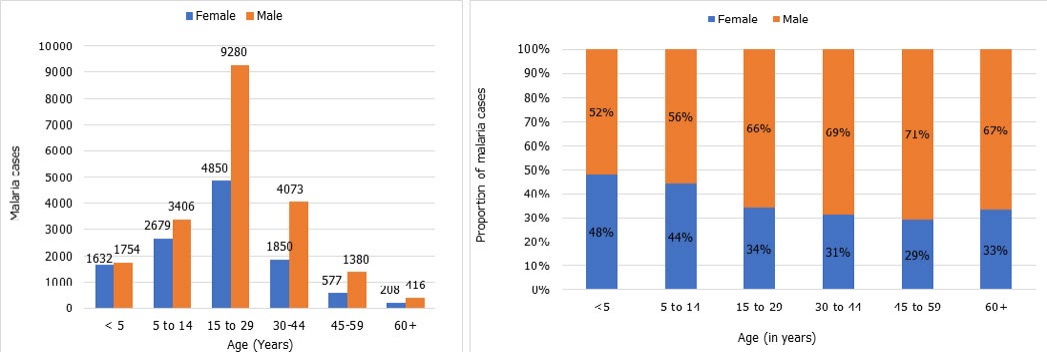
average malaria cases distribution by age groups and sex in Zanzibar

**Figure 2:**
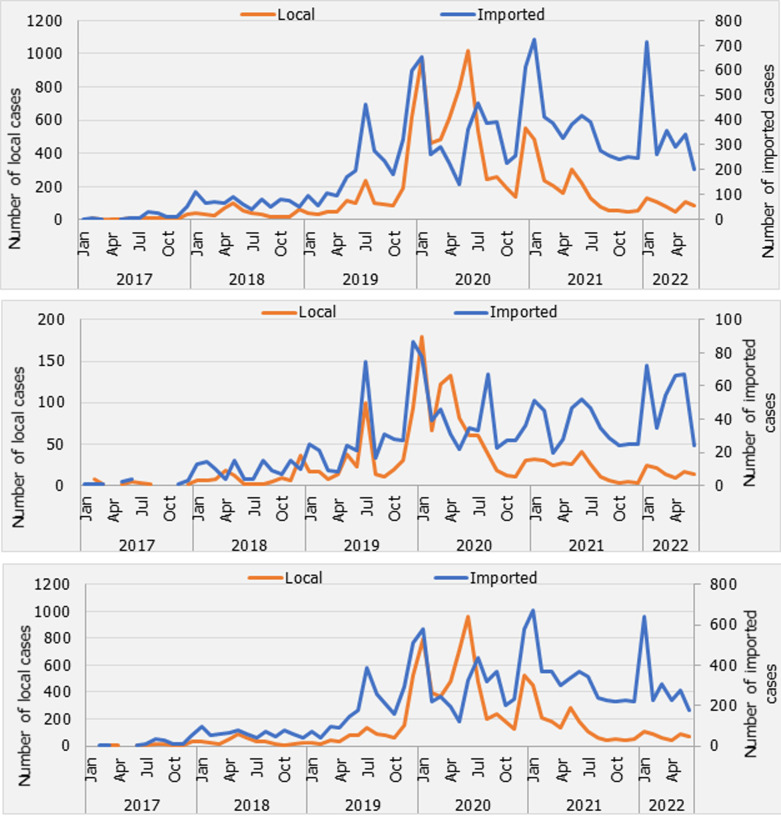
malaria cases classification in Zanzibar (A), Pemba (B), Unguja (C) 2017-2022

The annual parasite incidence (API) was found to have increased steadily over the last 5 years, from 2.7 (in 2017) to 3.6 (2021) cases per 1,000 population (Table 1). An epidemic was recorded in 2020 when the API sharply rose to 8.5 per 1,000 population. Similarly, malaria cases fluctuated but generally increased over the years. There has been an evident difference in the API between the two islands of Pemba and Unguja. In Pemba, the API was constantly below 2 cases per 1,000 population except during the 2020 epidemic year, while it was largely above 4 cases per 1,000 with a three times higher peak (compared to the lowest API in 2021) during the 2020 epidemic year (Table 1). Among all the districts in Zanzibar: Kati, Magharibi B and Mjini in Unguja and Wete and Micheweni in Pemba had higher API (Table 2) and were more affected by the epidemic wave in 2020. Young males between 15-29 years represented about three-quarters of the infections compared to the females of the same age group (Figure 1). The older age groups maintained the same proportion of males versus females but with a much lower incidence. About 68% of the detected malaria cases were imported into Zanzibar. The proportion of imported and indigenous malaria cases changed over time; indigenous cases were predominant during seasonal transmission peaks and during an epidemic event (Figure 2). Generally, there were fewer malaria cases in Pemba than in Unguja; however, similar trends of local and imported malaria cases were noted between the islands (Figure 2).

**Table 1:** annual parasite incidence (per 1,000 population) and malaria cases in Zanzibar, 2017-2021

Year	Annual parasite incidence per 1000			Malaria cases		
	Zanzibar	Pemba	Unguja	Zanzibar	Pemba	Unguja
2017	2.7	1.2	3.5	4,190	627	3,563
2018	3.4	1.9	4.2	5,408	987	4,421
2019	4.2	1.4	5.6	6,783	780	6,003
2020	8.5	2.8	11.5	14,289	1,594	12,695
2021	3.6	0.9	5.0	6,172	520	5,652

**Table 2:** district level annual parasite incidence rate (expressed as cases per 1,000 population) in Pemba and Unguja islands 2017-2021

Island	District	2017	2018	2019	2020	2021	2022
**Unguja**	Kaskazini A	2.8	4.2	4.8	7.6	3.3	1.7
	Kaskazini B	3.7	5.2	4.6	7.2	3.6	1.8
	Kati	8.8	9.4	11.9	18.1	11.6	5.3
	Kusini	6.6	5.0	4.6	9.3	9.6	2.6
	Magharibi A	2.2	3.7	4.8	6.4	4.1	1.9
	Magharibi B	3.2	3.7	5.7	10.2	5.9	2.6
	Mjini	2.9	4.6	7.4	16.5	4.4	1.6
**Pemba**	Mkoani	0.7	1.1	1.5	1.7	0.7	0.4
	Wete	1.2	2.0	2.2	4.0	1.6	1.2
	Chakechake	1.0	1.7	1.5	2.4	1.8	0.8
	Micheweni	3.1	4.0	2.5	4.5	0.9	0.7

**Figure 3:**
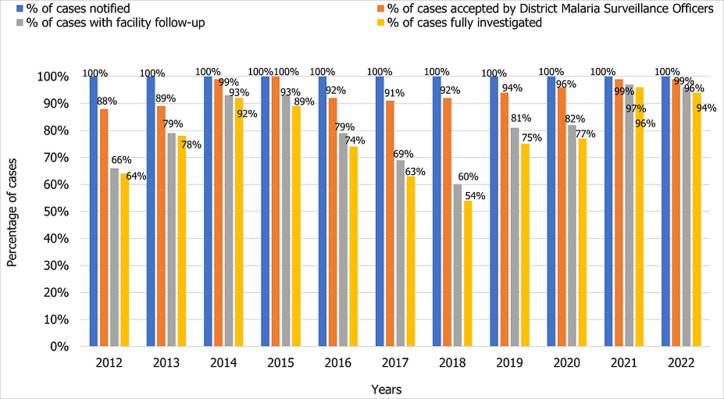
performance charts: annual cases notified, with health facility complete and fully investigated by the District Malaria Surveillance Officers (DMSO), 2012-2022

**Figure 4:**
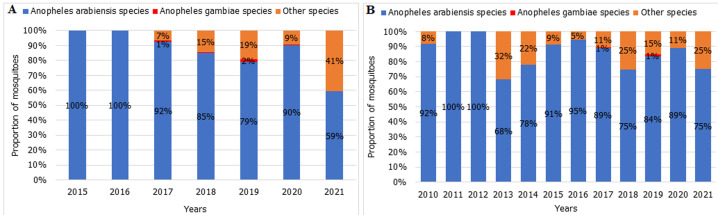
annual proportion of Anopheles species analyzed by PCR in Unguja (A) and Pemba (B) 2010-2021

**Figure 5:**
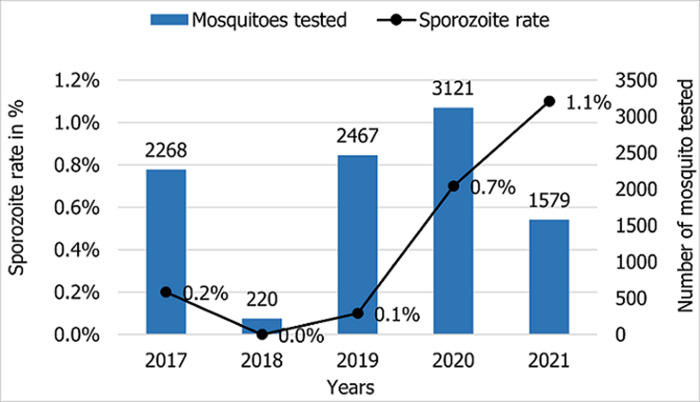
average sporozoite rate in Zanzibar between 2017 and 2021

The Zanzibar malaria surveillance system reports through national health information based on DHIS2, and the short message service-based Malaria Case Notification system (MCN) has gotten increasingly stronger and more efficient over the years. In 2017, of all the identified and notified malaria cases only 69% of cases were followed up, and 62% of cases were fully investigated (Figure 3). By 2021, case follow-up and investigation increased to 96% and 94%, respectively. *An. Arabiensis* remains the most prevalent Anopheles mosquito species in Pemba and Unguja (Figure 4). A few samples of *An. gambiae s.s*. were detected in 2019 but none in 2020 nor in 2021. Other mosquito species identified in the islands although in very little proportions (<2%) were *An. merus, An. coustani* and members of *An. funestus* group including *An. leesoni, An. rivulorum*, An. parensis and *An. funestus*. The sporozoite rate has been below 0.2% for most of the years, followed by a sharp rise to 0.7% in 2020, reaching a 1.1% peak in 2021 (Figure 5). A total of 1,693,722 LLINs were distributed in Zanzibar between 2018 and 2021 through different channels (Table 3). The number of LLINs increased more than three times over the past 5 years. The highest number of LLINs has been distributed through a mass replacement campaign (MRC) in 2020/21.

**Table 3:** long-lasting insecticidal nets distributed in Zanzibar from 2012-2021 by distribution channel

Year	Community	MRC	RCH	ZUCC	Total
2012				644,324	644,324
2016				631,605	631,605
2018	72,678		105,394		178,072
2019	117,549		104,825		222,374
2020	117,731	197,031	100,250		415,012
2021	69,151	712,872	96,241		878,264
**Total**	37,7109	909,903	406,710	1,275,929	2,969,651

MRC=Mass replacement campaign; RCH=Reproductive and child health; ZUCC=Zanzibar universal coverage campaign

There were consistent variations between budgeted, actual and anticipated funding activities, with an overall funding gap of about 12% and annual fluctuation of up to 40%. Almost three-quarters of the allocations were dedicated to vector control (50%) and case management (25%) activities. Global fund to fight aids tuberculosis and malaria and PMI financed the national malaria strategic plan by 56.6% and 41.6%, respectively, with wide variations between the allocated budget by thematic area. The review noted the difficulties in obtaining data on government contribution towards the malaria program but acknowledged significant contributions through counterpart funding, salaries of health workers, and other indirect contributions to the program. The allocation to health in the country’s budget increased 3.7 folds from 31.7 million USD (2017/18) to 117.3 million USD (2022/23). The total aggregate allocation to health increased from 6.7% in 2018/19 to 11.0% in 2022/23 of the total government budget. Nevertheless, the proportion of allocation for malaria program out of the total national health budget was generally less than 1% (0.27%- 2018/19, 0.28%-2019/20, 0.67%-2020/21, 0.36%-2021/22 and 0.19%-2022/23.

## Discussion

This assessment provided insight into the progress made in the implementation of malaria elimination activities in Zanzibar. The review noticed a steady increase in annual parasite incidence and malaria cases on the island between 2017 and 2021, with considerable heterogeneity between and within the two islands of Zanzibar. The API and malaria cases increased in Unguja but remained relatively stable in Pemba except during the 2019-2020 epidemic wave. Ecological conditions make Zanzibar a malaria-prone area; malaria positivity ranging between 20-40% has been recorded repeatedly in the past [11, 12]. Zanzibar had grappled with malaria for decades up until mid-2000 when the prevalence reached its historically lower level (below 1%) and succeeded in maintaining it low in the following 5 years [12]. Understanding the changing transmission dynamics in the remaining infection ‘hotspots’ is key. During the implementation of the ending NSP, an explosive epidemic was experienced in both Unguja and Pemba (2019-2020). While there was a general rise of cases throughout Zanzibar, Wete and Micheweni districts in Pemba and Kati, Magharibi B and Mjini districts in Unguja, were more affected. The reasons for the observed epidemic remain largely unknown. However, the contemporary upsurge of the outbreak and the COVID-19 pandemic should be further explored.

Zanzibar reported its first case of COVID-19 on March 18, 2020, immediately after the peak of the epidemic wave. The ZAMEP recorded reduced tendencies of patients´ attendance in health facilities for malaria services between 2019 and 2020. Symptomatic patients were uncomfortable attending the facilities because they would have been checked for COVID-19 with eventual isolation if confirmed positive. The untreated carriers may have sustained malaria transmission in the communities, spiking to the recorded levels. Disruption of malaria services due to the COVID-19 pandemic has been documented in many malaria-endemic settings [1]. World Health Organization estimated an excess of 10 cases per 1000 population at risk and 47000 deaths due to malaria service disruptions during the COVID-19 pandemic [1]. Specifically in the United Republic of Tanzania, the WHO recorded a 53% reduction in the number of malaria tests performed between April to June 2019 and the same period in 2020 attributable to the pandemic [1].

The analysis of the malaria data by sex and age noted that young male adults (15-29 years) are more largely affected than females and other age groups. This has been anecdotally associated with outdoor activities and behaviour of males, including recreational activities that expose them to early infectious mosquito bites resulting in malaria infection [13,14]. Elsewhere, it has been reported that asymptomatic infections clear naturally faster in females than males, thus making malaria more prevalent among children and adolescent males than females in the communities [14]. Zanzibar is a very low malaria risk setting, making it unlikely that females would harbour infections for longer to be cleared by natural immunity. Other factors responsible for the observed differences in malaria incidence between young males and females may need to be uncovered.

The proportion of imported and indigenous classified malaria cases changed over time. It should be noted that in the advanced stages of the 2019-2020 malaria outbreak, the predominant case classification switched from imported to indigenous cases. most probably due to: a) re-introduced malaria cases, b) disrupted access to health services, and c) limited mobility consequent to the COVID-19 pandemic. Nevertheless, on average between 2018 and 2022, slightly more than two-thirds of the identified malaria cases were classified as imported in Zanzibar, mainly from the mainland. Malaria in mainland Tanzania is very much heterogenous but on average moderate to high transmission levels [15]. The increased risk of malaria importation in the islands has been associated with the high mobility of the populations between Zanzibar and mainland Tanzania [16-19]. Using mobile phone data, previous studies have estimated an influx of between one and 12 imported infections per 1,000 people per year [16].

Given the differences in the malaria transmission levels between Zanzibar and all the neighbouring areas, including Kenya and mainland Tanzania; considering the ongoing mobility of people, malaria importation will likely be an ongoing challenge to the elimination attempts in the islands [19]. Epidemiological surveillance is supported by a pool of District Malaria Surveillance Officers employed by the MoH in each district. The assessment noted a robust malaria surveillance system with commendable levels of case follow-up and investigation (94% and 96%, respectively). The missed 5% of the cases may likely impart some unknown effect on mounting appropriate response interventions that cannot be underestimated. Moreover, it should be noted that in classifying malaria cases, whether indigenous or imported, the district malaria surveillance officers (DMSO) under the ZAMEP depend on patients´ travel history [20]. Travel history may likely have recall and interpretation biases from the interviewed patients and DMSOs. Strengthening molecular surveillance to aid in the routine classification of cases may be important to: 1) validate the existing system, and 2) quantify the actual extent of malaria importation and understand the patterns that are key in curbing the problem [21].

The assessment noted a predominance of *Anopheles arabiensis* in the Pemba and Unguja Islands. Malaria in Tanzania is transmitted chiefly by *Anopheles gambiae s.s, An. Arabiensis* and *An. Funestus* [22,23]. While the mosquito species are found sympatrically in many parts of the country, *An. Arabiensis* is the most adaptive mosquito species owing to its behavioural flexibility that increases its survival against effective indoor control interventions [24-26]. It is therefore not surprising that, in Zanzibar, previous studies reported a shift in the proportion of mosquito species collected to more *An. Arabiensis* following intensified interventions [11]. Consistent with studies done in the near past, sporozoite rate in Anopheles vectors from the Island are generally very low [27].

The principal vector control interventions in Zanzibar include IRS and LLINs. The assessment recorded some 1.7 million LLINs to have been distributed, almost doubling the number of nets distributed each year from 2018 to 2021. On the other hand, IRS is deployed as a reactive malaria vector control intervention, responding to increased cases in transmission hotspots. Despite low transmission levels, malaria risk perception is still high among the Zanzibaris, which guarantees the usage of LLINs and other preventive measures [28]. However, it should be noted that, as long as infectious mosquito species exist in Zanzibar, the outdoor feeding tendencies and prevailing densities make *An. arabiensis* a potential threat to malaria elimination by sustaining residual transmission. Therefore, the exploration of other outdoor vector control approaches is necessary. Pilot application of biolarvicides using drone technology shows potential for managing mosquito larval stages in extensive breeding habitats in Zanzibar [27]. Additionally, qualitative studies indicate that the Zanzibaris are considerably knowledgeable about malaria, its association with the environment and how the disease transmission can be reduced merely by modifying the environment [29]. Capitalising on this understanding of the community will likely take the Island a long way in the fight against malaria, vector- and other water-borne diseases. Anopheles stephensi is a potential threat to the elimination attempts in Zanzibar that cannot be ignored. The species originated in South East Asia and is spreading fast across Africa [30,31]. An stephensi is known for its high transmission efficiency, adaptation to domestic and peridomestic ecologies and, extremely high levels of insecticide resistance [31]. The recently modelled inter- and intra-continental maritime connectivity in Africa placed Tanzania among three countries (together with Mauritius and Kenya) at the highest risk of introduction [32]. There is a pressing need to intensify entomological surveillance on the Islands.

Programme financing is crucial to the implementation of activities and the roll-out of interventions. The review recorded an over-dependence of direct malaria expenditure on funding from international development partners, with an overall funding gap of 12% for the ending strategic plan. WHO estimates that reduced funding for malaria has greatly impacted the implementation of national strategic plans in many endemic countries [6]. For this, the WHO advocates for interventions tailored to local needs to optimise the already scarce resources. Nevertheless, donor support will almost always not match the country’s priorities and needs. It is gratifying to note that the allocation to health in the country budget increased 3.7 folds, and the total aggregate allocation to health increased by over 60% between 2017/18 and 2022/23. The review noted considerable fluctuation in the proportion of allocation for malaria out of the total national health budget over the 5 years and generally less than 1%. As health remains the priority sector in Zanzibar and the annual national budget is yet to reach the Abuja Declaration mark of 15%, the government must increasingly and strategically mobilise its resources to fund the deployment of malaria elimination interventions.

## Conclusion

The elimination of malaria in Zanzibar has been a long-standing moving target, yet achievable. Notwithstanding the implementation challenges, significant progress has been made in both islands in reducing the malaria prevalence and maintaining very low levels (i.e., <1%) for over a decade. The well-established case-based surveillance with immediate case notification and classification is key in the timely and appropriate identification of transmission ‘hotspots’ to trigger further investigation and response. The substantial difference in the transmission levels between and within the islands suggests a need for strategic re-orientation of the elimination attempts from a national-wide to a sub-national agenda. This will warrant the deployment of exclusive elimination and elimination and control interventions in districts depending on the sub-national level transmission risk, thereby optimising the available resources. The ongoing malaria transmission in the islands and, subsequently the review has indicated a weak multisectoral integration of malaria programming that has resulted in lost opportunities for greater gains in the fight against malaria. Imported malaria is becoming increasingly important in sustaining transmission in Zanzibar. Further work needs to be done to understand better the importation problem and the dynamics and determinants of population and ecological vulnerability on the Island to mitigate the effect of imported cases adequately. We recommend strengthening and sustaining mechanisms for the multisectoral, private sector, and higher-level partnership engagement. We also suggest that through multisectoral initiatives, malaria stakeholders should solicit more opportunities for mobilizing domestic resources to support the optimal elimination of malaria in Zanzibar.
